# The involvement of language‐associated networks, tracts, and cortical regions in frontotemporal dementia and amyotrophic lateral sclerosis: Structural and functional alterations

**DOI:** 10.1002/brb3.3250

**Published:** 2023-09-11

**Authors:** Marlene Tahedl, Ee Ling Tan, Rangariroyashe H. Chipika, Jasmin Lope, Jennifer C. Hengeveld, Mark A. Doherty, Russell L. McLaughlin, Orla Hardiman, Siobhan Hutchinson, Mary Clare McKenna, Peter Bede

**Affiliations:** ^1^ Computational Neuroimaging Group (CNG), School of Medicine Trinity College Dublin Dublin Ireland; ^2^ Smurfit Institute of Genetics Trinity College Dublin Dublin Ireland; ^3^ Department of Neurology St James's Hospital Dublin Ireland

**Keywords:** amyotrophic lateral sclerosis, frontotemporal dementia, language, MRI

## Abstract

**Background:**

Language deficits are cardinal manifestations of some frontotemporal dementia (FTD) phenotypes and also increasingly recognized in sporadic and familial amyotrophic lateral sclerosis (ALS). They have considerable social and quality‐of‐life implications, and adaptive strategies are challenging to implement. While the neuropsychological profiles of ALS–FTD phenotypes are well characterized, the neuronal underpinnings of language deficits are less well studied.

**Methods:**

A multiparametric, quantitative neuroimaging study was conducted to characterize the involvement of language‐associated networks, tracts, and cortical regions with a panel of structural, diffusivity, and functional magnetic resonance imaging (MRI) metrics. Seven study groups were evaluated along the ALS–FTD spectrum: healthy controls (HC), individuals with ALS without cognitive impairment (ALSnci), *C9orf72*‐negative ALS–FTD, *C9orf72*‐positive ALS–FTD, behavioral‐variant FTD (bvFTD), nonfluent variant primary progressive aphasia (nfvPPA), and semantic variant PPA (svPPA). The integrity of the Broca's area, Wernicke's area, frontal aslant tract (FAT), arcuate fascicle (AF), inferior occipitofrontal fascicle (IFO), inferior longitudinal fascicle (ILF), superior longitudinal fascicle (SLF), and uncinate fascicle (UF) was quantitatively evaluated. The functional connectivity (FC) between Broca's and Wernicke’ areas and FC along the FAT was also specifically assessed.

**Results:**

Patients with nfvPPA and svPPA exhibit distinctive patterns of gray and white matter degeneration in language‐associated brain regions. Individuals with bvFTD exhibit Broca's area, right FAT, right IFO, and UF degeneration. The ALSnci group exhibits Broca's area atrophy and decreased FC along the FAT. Both ALS–FTD cohorts, irrespective of *C9orf72* status, show bilateral FAT, AF, and IFO pathology. Interestingly, only *C9orf72*‐negative ALS–FTD patients exhibit bilateral uncinate and right ILF involvement, while *C9orf72*‐positive ALS–FTD patients do not.

**Conclusions:**

Language‐associated tracts and networks are not only affected in language‐variant FTD phenotypes but also in ALS and bvFTD. Language domains should be routinely assessed in ALS irrespective of the genotype.

## Graphical abstract

ALS patients without cognitive impairment exhibit Broca's area atrophy and decreased functional connectivity along the frontal aslant tract. ALS–FTD cohorts, irrespective of C9orf72 status, exhibit bilateral frontal aslant tract, arcuate fascicle, and inferior occipitofrontal fascicle pathology. Language‐associated tracts and networks are not only affected in language‐variant FTD phenotypes, but also in ALS and bvFTD.

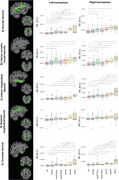

## 1 INTRODUCTION

The clinical profile of amyotrophic lateral sclerosis (ALS) is classically associated with bulbar dysfunction, respiratory weakness, decline in dexterity, and gait impairment (Yunusova et al., 2019). A substantial body of evidence from neuropsychology, neuroimaging, and postmortem studies has demonstrated varying degrees of frontotemporal involvement (Burke, Elamin, et al., 2016; Chipika, Christidi, et al., 2020; Christidi et al., 2019; McKenna, Corcia, et al., 2021), and the clinical overlap between ALS and frontotemporal dementia (FTD) has been cemented by the discovery of shared genetic variants such as hexanucleotide repeat expansion in *C9orf72* (Li Hi Shing, McKenna, et al., 2021). The spectrum of neuropsychological manifestations in ALS is often thought to be dominated by executive dysfunction and behavioral impairment, but the high prevalence of memory impairment, apathy, deficits in social cognition, and language impairment are increasingly recognized, further supporting the concept of an ALS–FTD spectrum (Burke, Pinto‐Grau, et al., 2016). A number of staging approaches have been proposed to stratify patients with ALS based on cognitive, behavioral, disability, pathological, and imaging criteria (Balendra et al., 2014; Brettschneider et al., 2013; Chiò et al., 2019; Elamin et al., 2017; Muller et al., 2016; Strong et al., 2009), recognizing the fundamental heterogeneity of the disease and helping to designate smaller, more homogenous subgroups.

Language impairment in ALS is increasingly recognized (Pinto‐Grau et al., 2018). It has considerable quality‐of‐life ramifications and may impact employment, understanding of information, enunciating care preferences, social interactions, and engagement with support providers. Language impairment may lead to social withdrawal, low mood, and depression and may potentially impact participation in clinical trials. While numerous well‐validated instruments exist to evaluate specific language domains, a number of confounding factors need to carefully taken into account in ALS for the accurate interpretation of performance of neuropsychological batteries. These include (1) disease‐specific factors such as hypercapnia, fatigue, anticholinergic medications for secretions, analgesia, poor‐quality sleep, and dysarthria; (2) concomitant neuropsychological deficits in other domains such as apathy and depression; and (3) extraneous/antecedent factors such as education and cognitive reserve (Costello et al., 2021). The complexity of linking neuropsychological manifestations to cerebral pathology is well recognized and a number of opinion papers highlighted the pitfalls to seek direct clinicoradiological associations (Verstraete et al., 2015). An alternative approach to evaluate disease burden patterns is the study of well‐defined, clinically stratified patient populations with quantitative neuroimaging and the interrogation of cerebral regions and networks physiologically associated with language function (Friederici, 2015; Galantucci et al., 2011; Middlebrooks et al., 2017). Accordingly, the main objective of this study is the detailed assessment of cerebral regions and networks physiologically linked to language domains and the comprehensive assessment of specific phenotypes along the ALS–FTD spectrum.

## 2 METHODS

### 2.1 Standard protocol approvals, registrations, and patient consents

All participants gave informed consent in accordance with the Ethics Approval of this research project (Beaumont Hospital, Dublin, Ireland—IRB).

### 2.2 Participants and neuroimaging

A total of 297 participants—184 patients and 113 healthy controls (HC)—were included into this study. The patient populations (Table 1) consisted of cognitively not impaired individuals with ALS (“ALSnci”; *N* = 99), ALS–FTD patients not carrying the hexanucleotide repeat expansion in *C9orfF72* (“ALSFTDC9–”; *N* = 29), ALS–FTD patients carrying the repeat expansion in the *C9orf72* (“ALSFTDC9+”; *N* = 24), behavioral‐variant FTD patients (“bvFTD”; *N* = 10), patients with nonfluent variant PPA (“nfvPPA”; *N* = 14), and semantic variant PPA (“svPPA”; *N* = 7). The El Escorial and Rascovsky criteria were used for the ALS–FTD cohort (Brooks et al., 2000; Rascovsky et al., 2011). Subjects with prior neurovascular syndromes, established psychiatric diagnoses, prior neurosurgery, or an inability to tolerate magnetic resonance imaging (MRI) scanning were excluded. Basic demographic and clinical data were carefully recorded for each participant on the day of magnetic resonance (MR) scanning (age, sex, handedness, years of education, medications, body region of symptom onset, family history of ALS or FTD). All individuals with ALS were screened for hexanucleotide repeat expansions by repeat‐primed PCR and tested for a panel of ALS‐associated genetic variants as described previously (Kenna et al., 2013). MR data were acquired on the same 3 Tesla Philips Achieva platform. For clinical interpretation, fluid‐attenuated inversion recovery (FLAIR) images were individually reviewed for each participant to identify unsuspected neurovascular or neuroinflammatory conditions. FLAIR imaging took place in axial orientation using an inversion recovery turbo spin echo sequence with the following imaging parameters: repetition time (TR)/echo time (TE) = 11,000/125 ms, inversion time (TI) = 2800 ms, field of view (FOV) = 230 × 183 × 150 mm, spatial resolution = 0.65 × 0.87 × 4 mm, 120° refocusing pulse, with flow compensation and motion smoothing, and a saturation slab covering the neck region. Three different MR modalities were acquired for quantitative data interpretation: structural T1‐weighted (T1w) imaging, resting‐state functional MRI (rs‐fMRI), and diffusion‐weighted imaging (DWI). Some patients could not tolerate the duration of the entire protocol (Table 1); therefore, not all three modalities were available for each patient. T1w data were acquired with a three‐dimensional inversion recovery‐prepared spoiled gradient‐recalled echo sequence with the following imaging parameters: FOV = 256 × 256 × 160 mm, 160 sagittal slices with no interslice gap, flip angle (FA) = 8°, voxel resolution (VR) = 1 mm isotropic, SENSE factor = 1.5, TR/TE = 8.5/3.9 ms, and TI = 1060 ms. DWI data were acquired with a spin‐echo echo planar imaging pulse sequence using a 32‐direction Stejskal–Tanner diffusion encoding scheme with *b*‐values of 0/1100 s/mm^2^ to record DWI data with the following settings: FOV = 245 × 245 × 150 mm, 60 axial slices with no interslice gaps, FA = 90°, VR = 2.5 mm isotropic, SENSE factor = 2.5, TR/TE = 7639/59 ms, dynamic stabilization, and spectral presaturation with inversion recovery fat suppression. To investigate fluctuations of the blood‐oxygen‐level‐dependent signal for functional imaging, an echo‐planar imaging sequence was utilized. A total of 220 volumes were acquired with eyes closed implementing the following parameters: FOV = 233 × 233 × 120 mm, 30 axial slices with no interslice gap, FA = 90°, VR = 2.875 × 2.875 × 4 mm isotropic, SENSE factor = 2.5, TR/TE = 2000/35 ms, acquisition time = 5 min and 41 s, and pixel bandwidth = ∼1900 Hz/Px (with slight variations of up to 150 Hz/Px between subjects).

**TABLE 1:** Demographic details of the study population.

	Patient subgroups								
							All patients	HC	*t*‐test (W)/chi‐square (C^2^)
	ALSnci	ALSFTDC9–	ALSFTDC9+	bvFTD	nfvPPA	svPPA			
Total number of subjects	99	29	24	10	15	7	184	113	NA
Complete T1w data sets	99	29	24	10	15	7	184	113	NA
Complete DWI data sets	94	29	22	10	13	5	173	111	NA
Complete fMRI data sets	96	26	22	7	8	2	161	111	NA
Age (years; mean ± *SD*)	58.73 ± 12.06	63.66 ± 11.13	55.50 ± 10.72	63.40 ± 6.43	71.33 ± 5.93	68.57 ± 6.29	60.74 ± 11.54	59.36 ± 10.66	W: *t*(251.47) = 1.04, *p* = .30
Sex, F/M	36/63	7/22	9/15	4/6	9/6	4/3	69/115	57/56	C^2^: *χ* ^2^(1, *N* = 297) = 4.29, *p* = .04^*^
Handedness, R/L	90/9	26/3	20/4	10/0	15/0	7/0	168/16	106/7	C^2^: *χ* ^2^(1, *N* = 297) = 0.31, *p* = .58
Years of education (years; mean ± *SD*)	13.62 ± 3.07	13.45 ± 4.21	14.04 ± 3.51	12.20 ± 4.18	13.00 ± 2.65	15.86 ± 2.20	13.60 ± 3.35	14.68 ± 3.52	W: *t*(228.11) = −2.61, *p* = .01^*^

Abbreviations: ALS, amyotrophic lateral sclerosis; ALSFTDC9–(+), (non)carriers of the hexanucleotide repeat expansion in *C9orf72*; ALSnci, cognitively not impaired ALS; bvFTD, behavioral variant FTD; DWI, diffusion‐weighted imaging; F, female; fMRI, functional MRI; FTD, frontotemporal dementia; HC, healthy control; L, left‐handed; M, male; MRI, magnetic resonance imaging; *N*, sample size; NA, not applicable; nfvPPA, nonfluent variant PPA; PPA, primary progressive aphasia; R, right‐handed; SD, standard deviation; svPPA, semantic variant PPA.

^a^ Welch two‐sample *t*‐tests were performed to test differences of age and years of education between all patients versus HC.

^b^ Chi‐square tests were performed to test differences of sex and handedness frequencies between all patients versus HC.

^*^Significant at an alpha‐level of *p* ≤ .05.

### 2.3 Cortical thickness estimation of Broca's and Wernicke's areas

**FIGURE 1:**
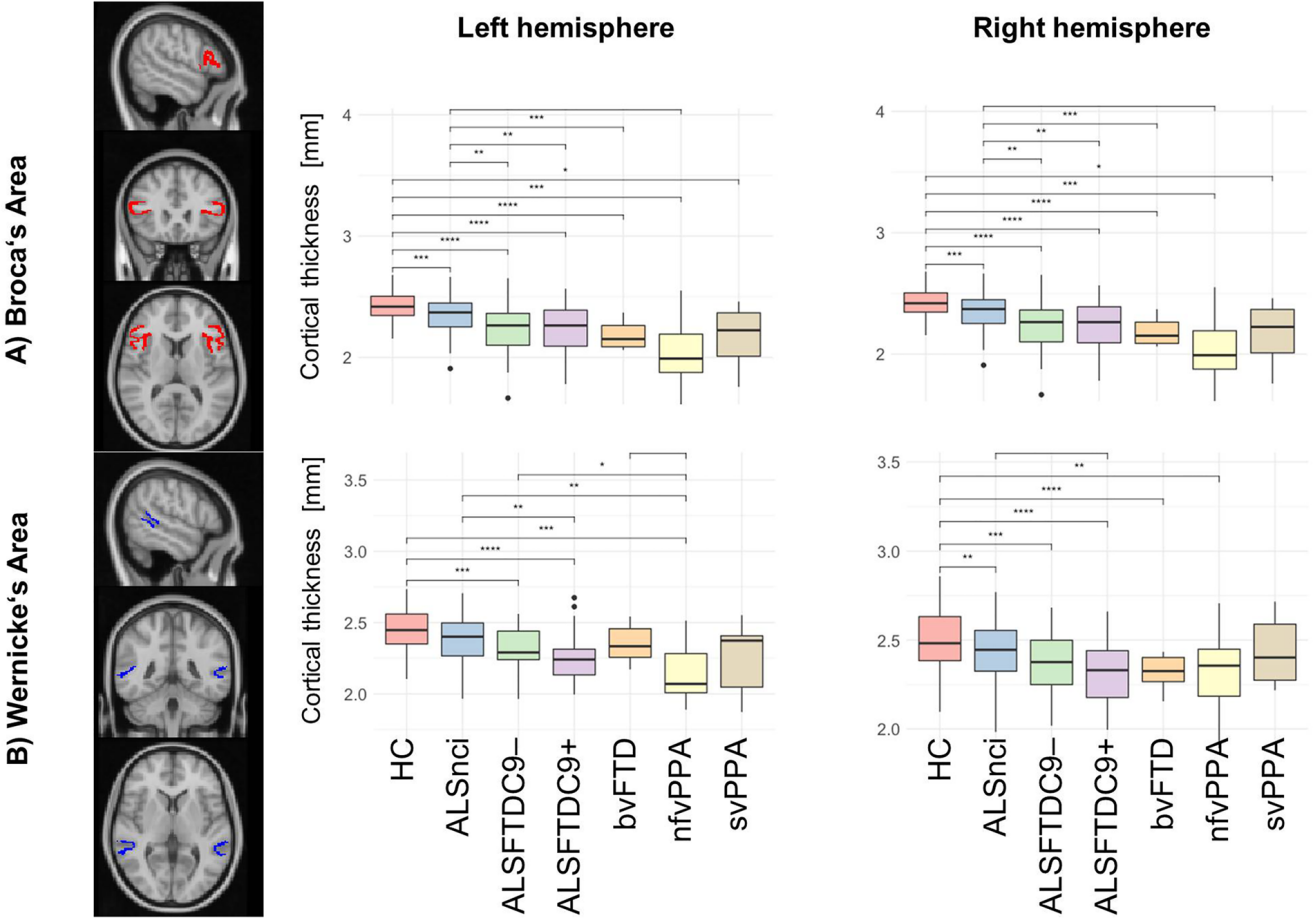
Cortical thickness (CT) changes in Broca's (a) and Wernicke's areas (b). Asterisks represent different levels of significance: +*p*_adj_ ≤ .05; *p_adj_ ≤ .01; and **p_adj_ ≤ .001. Box plots indicate medians and upper/lower quartiles, as well as outliers as dots. adj, adjusted; ALS, amyotrophic lateral sclerosis; ALSFTDC9–(+), (non)carriers of the repeat expansion in the *C9orf72* gene; ALSnci, noncognitively impaired ALS; ANOVA, analysis of variance; bvFTD, behavioral variant FTD; CT, cortical thickness; FTD, frontotemporal dementia; HC, healthy control; HSD, honest significant difference; MNI, Montreal Neurological Institute; nfvPPA, nonfluent variant PPA; PPA, primary progressive aphasia; ROI, region of interest; svPPA, semantic variant PPA.

Cortical thickness (CT) was estimated in Broca's and Wernicke's areas based on structural T1w data. T1w data were first preprocessed using FreeSurfer's (Fischl, 2012) automated “recon_all” pipeline, which—in brief—includes bias correction, brain extraction, normalization, and rendering of data onto a two‐dimensional surface representation of the cortex (Dale et al., 1999; Fischl et al., 1999). To facilitate data management for further downstream analyses, surface data were converted into “CIFTI” file format using Ciftify (Dickie et al., 2019), which uses tools from Workbench (Marcus et al., 2013). Additionally, the data were also preprocessed using the standard “fsl_anat” pipeline of FMRIB's Software Library (FSL) (Jenkinson et al., 2012). This, in brief, includes bias correction, brain extraction, and normalization to the MNI152 2 mm standard space by means of nonlinear registration, and outputs transformation matrices to be subsequently used for downstream image co‐registration of DWI and functional data. “Broca's area” was defined as the merged “pars opercularis” and “pars triangularis” labels of the Desikan–Killiany (DK) atlas (Desikan et al., 2006). Wernicke's area was defined based on the “banks of the superior temporal sulcus” of the DK atlas—encompassing parts of the superior temporal gyrus and posterior middle temporal gyrus, which is a topological approximation of Wernicke's area (Binder, 2015) (Figure 1). Language is lateralized to the left hemisphere in right‐handed subjects and also thought to be lateralized to the left in most left‐handed people (Buchsbaum et al., 2011; Szaflarski et al., 2002). However, we extracted Broca's and Wernicke's areas, that is, their contralateral right hemispheric analogues, to further evaluate interhemispheric differences. CT was defined as a single value for each region of interest (ROI) and subject, that is, the average of all vertices comprising that respective ROI.

### 2.4 Structural connectivity estimation of six language‐associated tracts

Structural connectivity (SC) was estimated based on diffusion‐weighted (DW) data. First, data were preprocessed using tools from MRtrix3 (Tournier et al., 2019), which included noise removal (Veraart et al., 2016), removal of Gibb's Ringing artifacts (Kellner et al., 2016), motion and eddy current corrections (Smith et al., 2004), as well as bias field correction (Tustison et al., 2010).

As integrity metrics of white tracts classically associated with language function were investigated, the following six fiber tracts were specifically assessed in each hemisphere: the arcuate fascicle (AF), inferior occipitofrontal fascicle (IFO), inferior longitudinal fascicle (ILF), superior longitudinal fascicle (SLF), uncinate fascicle (UF) (Friederici, 2015; Middlebrooks et al., 2017), as well as the frontal aslant tract (FAT), which connects the supplementary motor region/lateral superior frontal gyrus to the inferior frontal gyrus (La Corte et al., 2021). The segmentation pipeline TractSeg (Wasserthal et al., 2018) was used to identify AF, IFO, ILF, SLF, and UF, using a neural network algorithm to segment individual DWI data sets. TractSeg outputs three separate fiber bundles for SLF, which were merged into a single SLF map. The advantage of the neural‐network‐based method is that it does not assume anatomical commonality between subjects and detects individual white matter (WM) microstructure variations. The constrained spherical deconvolution (CSD) method was implemented to estimate fiber orientation distribution (fODF) at each voxel, and peaks of the spherical harmonic function were extracted using tools from MRtrix3 (Tournier et al., 2019). CSD is increasingly utilized instead of the tensor model to estimate fiber orientation and perform tractography since it may outperform the tensor model in regions of crossing fibers (Farquharson et al., 2013; Tournier et al., 2007, 2008). CSD requires the estimation of a so‐called “response function,” which we provided using the Dhollander method as implemented in MRtrix3. Even though DW data were only acquired with a *b*‐value of 1100 s/mm^2^, the additionally recorded *b* = 0 s/mm^2^ images allowed to implement a multishell approach nevertheless. Resulting fODFs were normalized according to Raffelt et al. (2017); spherical harmonic peaks were retrieved from the normalized measures, which then served as input values into TractSeg. This segmentation strategy could not be implemented in some subjects and tracts; therefore, the total number of subjects varied slightly between the tracts, as well as the relevant degrees of freedom (DOFs) as presented in Table 2. For the segmentation of the FAT, we first defined the source and target ROIs based on the available literature (La Corte et al., 2021), using the labels of the Glasser atlas (Glasser et al., 2016) in volumetric space. We aligned labels and DW images to the high‐resolution T1w data and calculated tractograms between each pair of ROIs using a probabilistic algorithm, generating 5000 streamlines per tract, using the analogue options and parameters for estimating fODF and ultimately tractography as for TractSeg. The 12 track images, six in each hemisphere, were then mapped onto track‐weighted images using the track density imaging method (Calamante et al., 2010), where each streamline contributes a value of unity to the final track‐weighted output map. We binarized this map using a threshold of a minimum of two streamlines per voxel. With the resulting binarized maps, SC could be estimated for each tract, which was defined as mean values of radial diffusivity (RD) and fractional anisotropy (FA) of all voxels comprising the respective tract. Both RD and FA are considered to reflect aspects of WM microstructure and are derivatives of the tensor model, which we calculated by means of least squares estimation (Veraart et al., 2013).

**TABLE 2:** Statistical comparisons of neuroimaging metrics between FTD patients versus healthy controls and post hoc comparisons among subject subgroups.

	One‐way ANOVA (omnibus test, main effect: “*diagnosis*”)			
	Left hemisphere		Right hemisphere	
	*F*‐value (DOF), *p*‐value	Significant pairwise contrasts (post hoc) [*p*‐value]	*F*‐value (DOF), *p*‐value	Significant pairwise contrasts (post hoc) [*p*‐value]
Cortical thickness				
Broca's area	*F*(6, 286) = 23.90, *p* < .001^*^	‐ HC vs. ALSnci [*p*_adj_ = .003] ‐ HC vs. ALSFTD_C9– [*p*_adj_ < .001] ‐ HC vs. ALSFTD_C9+ [*p*_adj_ < .001] ‐ HC vs. bvFTD [*p*_adj_ < .001] ‐ HC vs. nfvPPA [*p*_adj_ < .001] ‐ HC vs. svPPA [*p*_adj_ = .012] ‐ ALSnci vs. ALSFTD_C9– [*p*_adj_ = .023] ‐ ALSnci vs. ALSFTD_C9+ [*p*_adj_ < .001] ‐ ALSnci vs. bvFTD [*p*_adj_ = .032] ‐ ALSnci vs. nfvPPA [*p*_adj_ < .001]	*F*(6, 286) = 23.87, *p* < .001^*^	‐ HC vs. ALSnci [*p*_adj_ = .002] ‐ HC vs. ALSFTD_C9– [*p*_adj_ < .001] ‐ HC vs. ALSFTD_C9+ [*p*_adj_ < .001] ‐ HC vs. bvFTD [*p*_adj_ < .001] ‐ HC vs. nfvPPA [*p*_adj_ < .001] ‐ HC vs. svPPA [*p*_adj_ = .03] ‐ ALSnci vs. ALSFTD_C9– [*p*_adj_ = .014] ‐ ALSnci vs. ALSFTD_C9+ [*p*_adj_ < .001] ‐ ALSnci vs. bvFTD [*p*_adj_ = .010] ‐ ALSnci vs. nfvPPA [*p*_adj_ < .001]
Wernicke's area	*F*(6, 286) = 13.22, *p* < .001^*^	‐ HC vs. ALSFTD_C9– [*p*_adj_ = .005] ‐ HC vs. ALSFTD_C9+ [*p*_adj_ < .001] ‐ HC vs. nfvPPA [*p*_adj_ < .001] ‐ ALSnci vs. ALSFTD_C9+ [*p*_adj_ = .006] ‐ ALSFTD_C9– vs. nfvPPA [*p*_adj_ = .035] ‐ bvFTD vs. nfvPPA [*p*_adj_ = .033]	*F*(6, 286) = 9.10, *p* < .001^*^	‐ HC vs. ALSnci [*p*_adj_ = .023] ‐ HC vs. ALSFTD_C9– [*p*_adj_ = .010] ‐ HC vs. ALSFTD_C9+ [*p*_adj_ < .001] ‐ HC vs. bvFTD [*p*_adj_ = .020] ‐ HC vs. nfvPPA [*p*_adj_ = .004] ‐ ALSnci vs. ALSFTD_C9+ [*p*_adj_ = .007]
Structural connectivity: Frontal aslant tract (FAT)				
FA	*F*(6, 271) = 6.05, *p* < .001^*^	‐ HC vs. ALSFTD_C9+ [*p*_adj_ = .030] ‐ HC vs. nfvPPA [*p*_adj_ = .012] ‐ ALSnci vs. nfvPPA [*p*_adj_ = .036]	*F*(6, 271) = 5.78, *p* < .001^*^	‐ HC vs. ALSFTD_C9+ [*p*_adj_ = .023]
RD	*F*(6, 271) = 12.99, *p* < .001^*^	‐ HC vs. ALSFTD_C9– [*p*_adj_ < .001] ‐ HC vs. ALSFTD_C9+ [*p*_adj_ = .023] ‐ HC vs. nfvPPA [*p*_adj_ < .001] ‐ ALSnci vs. ALSFTD_C9– [*p*_adj_ = .017] ‐ ALSnci vs. nfvPPA [*p*_adj_ = .017] ‐ nfvPPA vs. bvFTD [*p*_adj_ < .001]	*F*(6, 271) = 11.95, *p* < .001^*^	‐ HC vs. ALSFTD_C9– [*p*_adj_ < .001] ‐ HC vs. ALSFTD_C9+ [*p*_adj_ = .011] ‐ HC vs. bvFTD [*p*_adj_ = .0281] ‐ HC vs. nfvPPA [*p*_adj_ < .001] ‐ ALSnci vs. ALSFTD_C9– [*p*_adj_ = .016] ‐ ALSnci vs. nfvPPA [*p*_adj_ = .002]
Structural connectivity: Arcuate fascicle (AF)				
FA	*F*(6, 282) = 5.50, *p* < .001^*^	‐ HC vs. ALSFTD_C9+ [*p*_adj_ = .006]	*F*(6, 282) = 2.38, *p* = .03^*^	–
RD	*F*(6, 271) = 21.85, *p* < .001^*^	‐ HC vs. ALSFTD_C9– [*p*_adj_ = .003] ‐ HC vs. ALSFTD_C9+ [*p*_adj_ < .001] ‐ HC vs. nfvPPA [*p*_adj_ < .001] ‐ HC vs. svPPA [*p*_adj_ = .012] ‐ ALSnci vs. ALSFTD_C9+ [*p*_adj_ = .032] ‐ ALSnci vs. nfvPPA [*p*_adj_ < .001] ‐ ALSnci vs. svPPA [*p*_adj_ = .001] ‐ ALSFTD_C9– vs. nfvPPA [*p*_adj_ < .001] ‐ ALSFTD_C9+ vs. nfvPPA [*p*_adj_ = .013] ‐ bvFTD vs. nfvPPA [*p*_adj_ = .008]	*F*(6, 282) = 9.89, *p* < .001^*^	‐ HC vs. ALSFTD_C9– [*p*_adj_ < .001] ‐ HC vs. ALSFTD_C9+ [*p*_adj_ < .001] ‐ HC vs. nfvPPA [*p*_adj_ < .001]
Structural connectivity: Inferior occipitofrontal fascicle (IFO)				
FA	*F*(6, 281) = 0.76, *p* = .60	NA	*F*(6, 280) = 1.13, *p* = .35	NA
RD	*F*(6, 281) = 9.35, *p* < .001^*^	‐ HC vs. ALSFTD_C9– [*p*_adj_ = .028] ‐ HC vs. ALSFTD_C9+ [*p*_adj_ = .018] ‐ HC vs. svPPA [*p*_adj_ = .003] ‐ ALSnci vs. svPPA [*p*_adj_ = .026]	*F*(6, 280) = 14.48, *p* < .001^*^	‐ HC vs. ALSFTD_C9– [*p*_adj_ = .001] ‐ HC vs. ALSFTD_C9+ [*p*_adj_ = .005] ‐ HC vs. bvFTD [*p*_adj_ = .030] ‐ HC vs. nfvPPA [*p*_adj_ = .010] ‐ HC vs. svPPA [*p*_adj_ = .003] ‐ ALSnci vs. svPPA [*p*_adj_ = .005]
Structural connectivity: Inferior longitudinal fascicle (ILF)				
FA	*F*(6, 278) = 0.70, *p* = .65	NA	*F*(6, 272) = 0.85, *p* = .53	NA
RD	*F*(6, 278) = 9.34, *p* < .001^*^	‐ HC vs. svPPA [*p*_adj_ < .001] ‐ ALSnci vs. svPPA [*p*_adj_ < .001] ‐ ALSFTD_C9– vs. svPPA [*p*_adj_ < .001] ‐ ALSFTD_C9+ vs. svPPA [*p*_adj_ < .001] ‐ bvFTD vs. svPPA [*p*_adj_ < .001] ‐ nfvPPA vs. svPPA [*p*_adj_ < .001]	*F*(6, 272) = 10.76, *p* < .001^*^	‐ HC vs. ALSFTD_C9– [*p*_adj_ = .045] ‐ HC vs. nfvPPA [*p*_adj_ = .046] ‐ HC vs. svPPA [*p*_adj_ < .001] ‐ ALSnci vs. svPPA [*p*_adj_ < .001] ‐ ALSFTD_C9– vs. svPPA [*p*_adj_ = .007] ‐ ALSFTD_C9+ vs. svPPA [*p*_adj_ = .012] ‐ bvFTD vs. svPPA [*p*_adj_ = .031]
Structural connectivity: Superior longitudinal fascicle (SLF)				
FA	*F*(6, 282) = 8.40, *p* = .04^*^	‐ HC vs. nfvPPA [*p*_adj_ < .001] ‐ ALSnci vs. nfvPPA [*p*_adj_ < .001] ‐ ALSFTD_C9– vs. nfvPPA [*p*_adj_ = .002]	*F*(6, 281) = 8.77, *p* < .001^*^	‐ HC vs. ALSFTD_C9+ [*p*_adj_ < .001] ‐ ALSnci vs. ALSFTD_C9+ [*p*_adj_ < .001] ‐ ALSFTD_C9– vs. ALSFTD_C9+ [*p*_adj_ < .001] ‐ ALSFTD_C9+ vs. bvFTD [*p*_adj_ = .050] ‐ ALSFTD_C9+ vs. nfvPPA [*p*_adj_ = .023]
RD	*F*(6, 282) = 2.24, *p* < .001^*^	–	*F*(6, 281) = 3.57, *p* = .002^*^	–
Structural connectivity: Uncinate fascicle (UF)				
FA	*F*(6, 270) = 1.08, *p* = .37	NA	*F*(6, 273) = 3.16, *p* = .005^*^	–
RD	*F*(6, 270) = 12.02, *p* < .001^*^	‐ HC vs. ALSFTD_C9– [*p*_adj_ < .001] ‐ HC vs. nfvPPA [*p*_adj_ = .029] ‐ HC vs. svPPA [*p*_adj_ < .001] ‐ ALSnci vs. ALSFTD_C9– [*p*_adj_ = .010] ‐ ALSnci vs. svPPA [*p*_adj_ < .001] ‐ ALSFTD_C9– vs. svPPA [*p*_adj_ = .041] ‐ ALSFTD_C9+ vs. svPPA [*p*_adj_ = .003] ‐ bvFTD vs. svPPA [*p*_adj_ = .029]	*F*(6, 273) = 21.43, *p* < .001^*^	‐ HC vs. ALSFTD_C9– [*p*_adj_ < .001] ‐ HC vs. bvFTD [*p*_adj_ < .001] ‐ HC vs. svPPA [*p*_adj_ < .001] ‐ ALSnci vs. ALSFTD_C9– [*p*_adj_ < .001] ‐ ALSnci vs. bvFTD [*p*_adj_ = .006] ‐ ALSnci vs. svPPA [*p*_adj_ < .001] ‐ ALSFTD_C9– vs. svPPA [*p*_adj_ < .001] ‐ ALSFTD_C9+ vs. svPPA [*p*_adj_ < .001] ‐ bvFTD vs. svPPA [*p*_adj_ < .001] ‐ nfvPPA vs. svPPA [*p*_adj_ < .001]
Functional connectivity				
Broca–Wernicke FC	*F*(6, 261) = 1.71, *p* = .12	NA	*F*(6, 261) = 1.22, *p* = .30	NA
Aslant tract FC	*F*(6, 261) = 3.41, *p* = .003^*^	‐ HC vs. ALSnci [*p*_adj_ = .001]	*F*(6, 261) = 1.54, *p* = .17	NA

*Note*: The symbol “–” represents that post hoc testing yielded no significant pairwise comparisons despite the overall significant ANOVA omnibus test.

Abbreviations: adj, adjusted; AI, artificial intelligence; ALS, amyotrophic lateral sclerosis; ALSFTDC9–(+), (non)carriers of the hexanucleotide repeat expansion in *C9orf72*; ALSnci, noncognitively impaired ALS; ANOVA, analysis of variance; bvFTD, behavioral variant FTD; DOF, degrees of freedom; DWI, diffusion‐weighted imaging; FA, fractional anisotropy; FC, functional connectivity; FTD, frontotemporal dementia; HC, healthy control; HSD, honest significant difference; NA, not applicable (here, ANOVA omnibus test was not significant and therefore post hoc testing was not justified); nfvPPA, nonfluent variant PPA; PPA, primary progressive aphasia; RD, radial diffusivity; rs‐fMRI, resting‐state functional magnetic resonance imaging; svPPA, semantic variant PPA; T1w, T1‐weighted.

^*^Significant at an alpha level of *p* ≤ .05.

### 2.5 Functional connectivity between Broca's and Wernicke's areas and along the FAT

To evaluate any potential discriminative utility of functional connectivity (FC) to differentiate patient groups, we estimated FC for two ROI pairs, namely, FC between Broca's and Wernicke's areas as well as FC along FAT (i.e., between the supplementary motor region/lateral superior frontal gyrus and the inferior frontal gyrus). FC was calculated for each subject in MNI152 2 mm standard space using FSL's feat pipeline, including brain extraction, slice–time correction, and motion correction. Correction of head‐motion‐related artifacts was added in a separate data preparation step using FSL's AROMA algorithm (Pruim et al., 2015). Each patient's preprocessed, de‐noised functional image was standardized to the MNI152 2 mm standard space for high‐level group comparisons in a two‐step procedure: (1) linear co‐registration of native high‐resolution data using 6 DOFs, followed by (2) nonlinearly warping into standard space using 12 DOFs. FC was then defined as Fisher *z*‐transformed Pearson's correlation between the mean time courses of the above specified ROIs, separately in the two hemispheres. FC was calculated FC in Matlab R2021b (The Mathworks), using CoSMoMVPA (Oosterhof et al., 2016) and FieldTrip (Oostenveld et al., 2011) toolboxes.

### 2.6 Statistical modeling

Statistical analyses were conducted with RStudio (R version 4.2.2). Differences in age and education between patient cohorts and HC were examined using Welch two‐sample *t*‐tests. Sex and handedness profiles were compared using chi‐square testing. To test differences in neuroimaging metrics between the patient cohorts and HC, a one‐way analysis of variance (ANOVA) omnibus test was implemented, correcting for the confounding effects of age, sex, handedness, and years of education. When the omnibus test was significant at an alpha level of *p* ≤ .05, post hoc pairwise differences of means were explored using Tukey's Honestly Significance Test (HSD) generating *p*‐values corrected for the family‐wise error rate. Our initial results suggested that RD may better discriminate between the subgroups compared to FA in terms of SC. To further test this, another chi‐square test was implemented comparing the frequencies of significant pairwise contrasts in Tukey's HSD tests (Table 2). Specifically, we compared the counts of all possible significant post hoc contrasts (21 subject group combinations × 6 tracts × 2 hemispheres = 252 possible contrasts) and observed significant post hoc contrasts for RD and FA, respectively.

### 2.7 Data availability

Group‐level outputs, post hoc statistics, and additional information on data processing pipelines can be requested from the corresponding author. Clinical and neuroimaging data for individual patients cannot be made available due to institutional regulations and departmental policies.

## 3 RESULTS

### 3.1 Demographics

Descriptive demographic data are summarized in Table 1. While age (*t*(251.47) = 1.04, *p* = .30) and handedness (*χ* ^2^(1, *N* = 297) = 0.31, *p* = .58) were adequately matched, the aggregated patient group differed from the HC group in terms of gender distribution (*χ* ^2^(1, *N* = 297) = 4.29, *p* = .04)—suggesting a higher relative amount of females across the patient subgroups—and years of education (*t*(228.11) = −2.61, *p* = .01), indicative of longer education in HC. For downstream statistical modeling, all four variables were accounted for to correct for their potential confounding effects. Individuals with ALS tested negative for a panel of ALS‐associated genetic variants (Kenna et al., 2013).

### 3.2 Cortical thickness

CT of Broca's area differed significantly between the groups (*F*(6, 286) = 23.90, *p* < .001), and post hoc testing revealed lower values of all six patient subgroups compared to controls (Figure 1a; Table 2). The ALSnci group exhibited higher values compared to most patient subgroups other than svPPA. Analogous results were found for this cortical region in the right hemisphere. Wernicke's area thickness (Figure 1b) also showed group differences (*F*(6, 286) = 13.22, *p* < .001) and HC displayed higher values versus most patient subgroups except ALSnci and bvFTD. NfvPPA patients exhibited lower values compared to other groups except ALSFTDC9+ and svPPA. For the equivalent region in the right hemisphere, lower values were identified in most patient groups (except svPPA) compared to HC.

### 3.3 Structural connectivity

**FIGURE 2:**
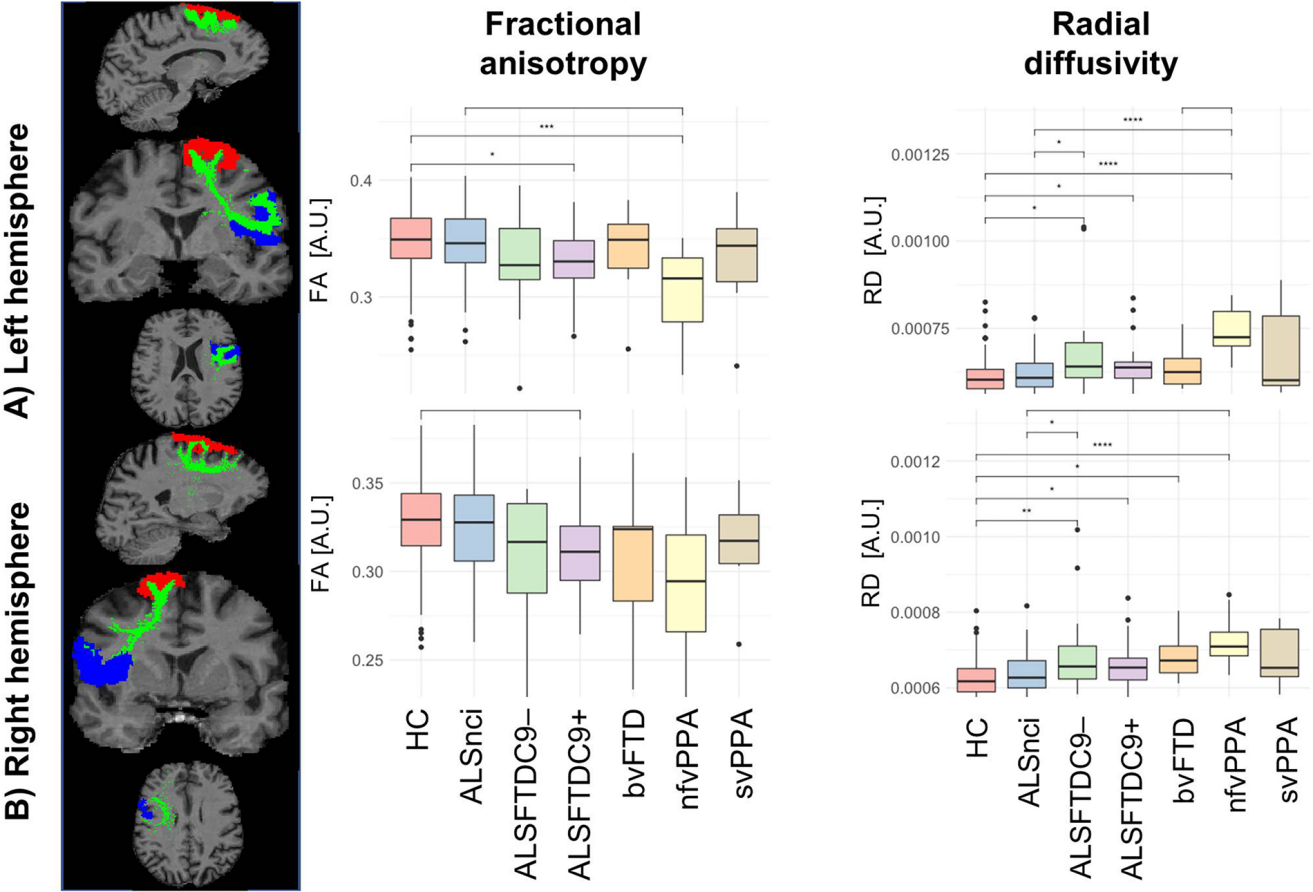
Structural connectivity (SC) alterations in the left (a) and right (b) frontal aslant tracts. Asterisks represent different levels of significance: +*p*_adj_ ≤ .05; *p_adj_ ≤ .01; and **p_adj_ ≤ .001. Box plots indicate medians and upper/lower quartiles, as well as outliers as dots. adj, adjusted; ALS, amyotrophic lateral sclerosis; ALSFTDC9–(+), (non)carriers of the repeat expansion in the *C9orfF72* gene; ALSnci, noncognitively impaired ALS; ANOVA, analysis of variance; bvFTD, behavioral variant FTD; FA, fractional anisotropy; FTD, frontotemporal dementia; HC, healthy control; HSD, honest significant difference; nfvPPA, nonfluent variant PPA; PPA, primary progressive aphasia; RD, radial diffusivity; ROI, region of interest; SC, structural connectivity; svPPA, semantic variant PPA.

**FIGURE 3:**
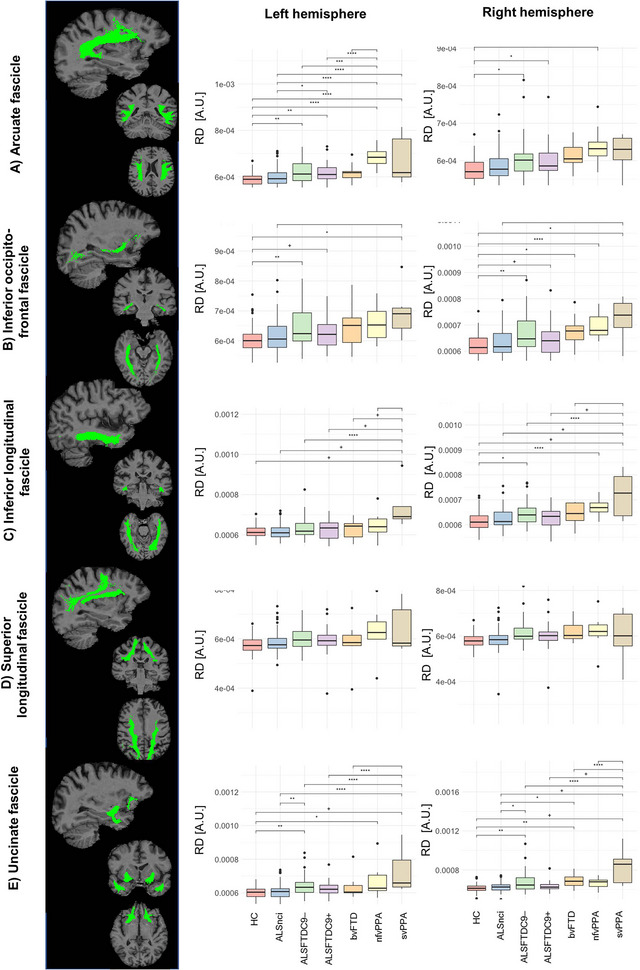
The structural connectivity (SC) alterations in language‐associated white matter tracts. Asterisks represent different levels of significance: +*p*_adj_ ≤ .05; *p_adj_ ≤ .01; and **p_adj_ ≤ .001. Box plots indicate medians and upper/lower quartiles, as well as outliers as dots. adj, adjusted; AF, arcuate fascicle ALS, amyotrophic lateral sclerosis; ALSFTDC9–(+), (non)carriers of the repeat expansion in the *C9orf72* gene; ALSnci, noncognitively impaired ALS; ANOVA, analysis of variance; bvFTD, behavioral variant FTD; FA, fractional anisotropy; FTD, frontotemporal dementia; IFO, inferior occipitofrontal fascicle; ILF, inferior longitudinal fascicle; HC, healthy control; HSD, honest significant difference; nfvPPA, nonfluent variant PPA; PPA, primary progressive aphasia; RD, radial diffusivity; ROI, region of interest; SC, structural connectivity; SLF, superior longitudinal fascicle; svPPA, semantic variant PPA; UF, uncinate fascicle.

SC of each language‐associated tracts showed group differences. RD captured more significant pairwise contrasts in post hoc testing. Statistical details are presented in Table 2, and the most significant findings are listed below. The FAT (Figure 2) was affected in most patient cohorts in both hemispheres compared to controls. The ALSnci group was the least affected patient group. NfvPPA patients were the most affected, followed by ALSFTDC9+. The AF was most affected in nfvPPA and, to a lesser degree, in ALSFTDC9+ patients. This was especially evident for RD and seemed specific for the left hemisphere (Figure 3a). Left‐hemispheric IFO was not affected in bvFTD, nfvPPA, and ALSnci compared to controls but affected in ALSFTDC9+/– and svPPA as measured by RD. The svPPA group tended to be the most affected patient group. Left‐hemispheric ILF of svPPA patients was the worst affected, since it showed significantly higher mean RD values versus all other groups in post hoc testing (Figure 3c). In the right hemisphere, the relative higher disease burden of svPPA was also evident but not as pronounced as on the left hemisphere. While RD did not vary for the SLF on neither hemisphere (Figure 3d), FA suggested that the nfvPPA group was the worst affected patient subgroup for the left hemisphere, yielding significantly lower values as compared to HC, ALSnci, and ALSFTDC9–. In the right hemisphere, however, the ALSFTDC9+ group was the most affected group, yielding lower values as compared to all other groups but svPPA. Finally, the UF was mostly affected in the svPPA group with higher RD as compared to most other subgroups and for both hemispheres (Figure 3e). Moreover, we observed that HC and ALSnci yielded comparable results and overall similar trends in post hoc testing. Across all tracts in both hemispheres, RD analyses yielded to more significant post hoc pairwise contrasts (*N* = 77) than FA (*N* = 13) (Table 2) (*χ* ^2^(1, *N* = 504) = 53.69, *p* < .001).

### 3.4 Functional connectivity

**FIGURE 4:**
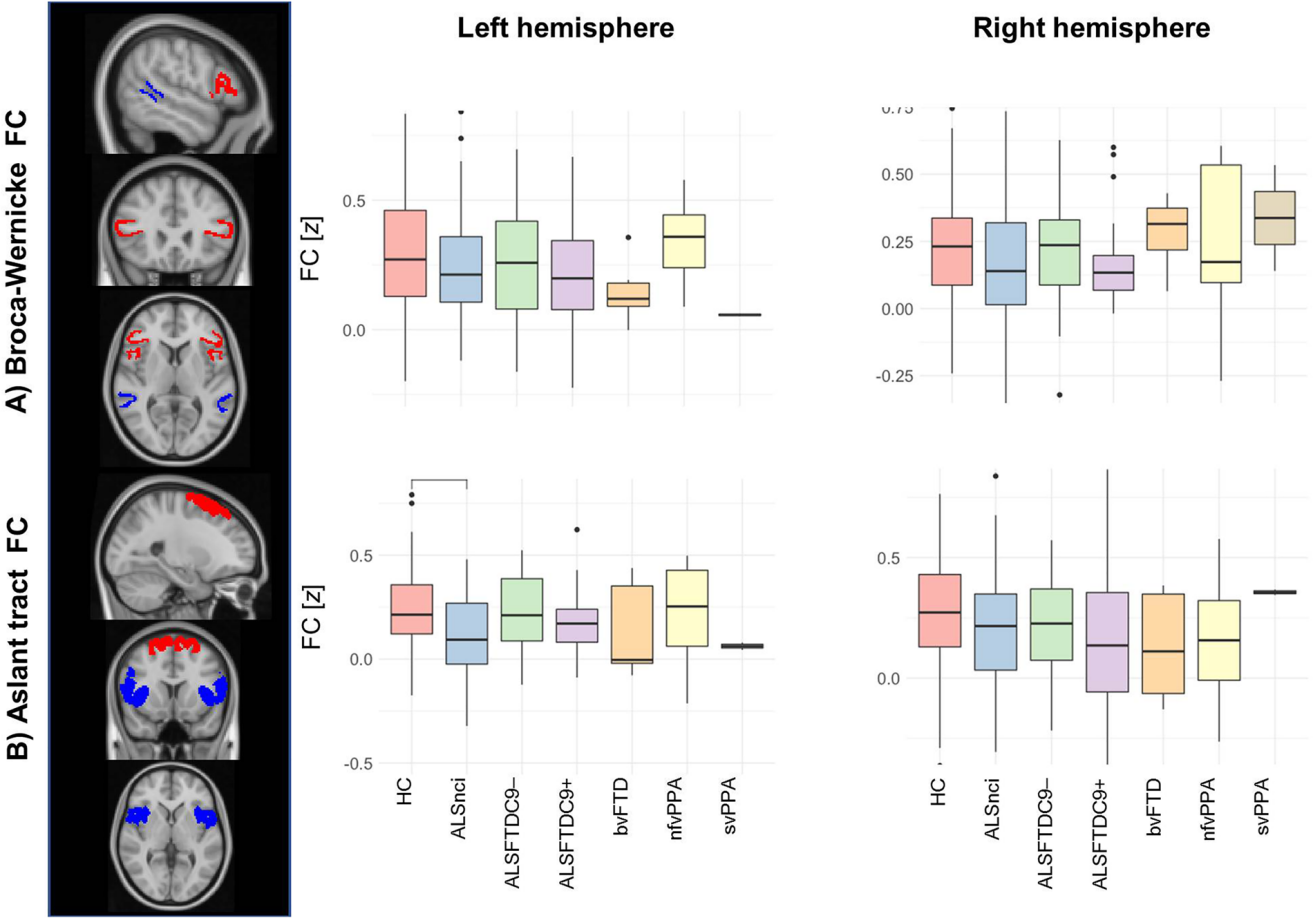
Functional connectivity (FC) alterations between Broca's and Wernicke's areas (a), and along the frontal aslant tract (b). Box plots indicate medians and upper/lower quartiles, as well as outliers as dots. ALS, amyotrophic lateral sclerosis; ALSFTDC9–(+), (non)carriers of the repeat expansion in the *C9orfF72* gene; ALSnci, noncognitively impaired ALS; ANOVA, analysis of variance; bvFTD, behavioral variant FTD; FA, fractional anisotropy; FTD, frontotemporal dementia; HC, healthy control; HSD, honest significant difference; MNI, Montreal Neurological Institute; nfvPPA, nonfluent variant PPA; PPA, primary progressive aphasia; RD, radial diffusivity; ROI, region of interest; svPPA, semantic variant PPA.

FC between Broca's and Wernicke's areas does not differentiate patient groups in either the left (*F*(6, 261) = 1.71, *p* = .12) or right hemisphere (*F*(6, 261) = 1.22, *p* = .30) (Figure 4a). FC along FAT does exhibit group differences (*F*(6, 261) = 3.41, *p* = .003), but the only significant pairwise contract post hoc is ALSnci exhibiting lower FC compared to controls (*p*_adj_ = .001). No similar pattern was identified in the right hemisphere (*F*(6, 261) = 1.54, *p* = .17) (Figure 4b).

## 4 DISCUSSION

Our results not only confirm the significant pathology of language‐associated brain regions in language‐variant FTD phenotypes (McKenna et al., 2022) but also showcase the pathology of these regions in ALS. Neuroimaging signatures in FTD are very well characterized in the literature (McKenna et al., 2023), but it is noteworthy that in our analyses, Wernicke's area is most affected in nfvPPA and unaffected in svPPA compared to controls. Patients with nfvPPA exhibited the lowest left hemispheric CT in both Broca's and Wernicke's areas. Similarly, the integrity of the FAT was the most affected in this group. Interestingly, our cohort of bvFTD exhibits bihemispheric Broca's area pathology and cortical involvement in the right‐hemispheric equivalent of Wernicke's area. From a tractography point of view, the bvFTD group also exhibits RD changes in the right FAT, right inferior IFO, and right uncinate compared to healthy controls.

The main finding of this study is the demonstration that ALS cohorts also show varying degrees of language region involvement depending on their genotype and phenotype. The gene‐negative ALSnci group shows bihemispheric Broca's area atrophy, as well as cortical thinning in the right‐hemispheric equivalent of Wernicke's area. Despite the detected cortical atrophy in ALSnci, the white matter tracts evaluated showed no degenerative change in this cohort compared to controls. However, ALSnci patients exhibit lower FC along the FAT than controls. Both ALS–FTD cohorts exhibit Broca's and Wernicke's area atrophy in the left hemisphere as well as in their contralateral equivalents. The *C9orf72*‐positive ALS–FTD cohort is the only ALS group with bilateral FA reduction along the FAT, but FAT RD is increased bilaterally in both ALS–FTD cohorts irrespective of *C9orf72* status. Both ALS–FTD groups show arcuate and inferior occipitofrontal pathology bilaterally based on RD. *C9orf72*‐positive ALS–FTD patients show additional right SLF involvement. Interestingly only the C9‐negative ALS–FTD group demonstrates bilateral uncinate and right ILF involvement, while C9‐positive ALS–FTD patients do not (Table 2; Figures 2, 3, 4).

Our analyses highlight that focal structural changes alone do not account for clinical symptoms and instead of solely performing voxel‐wise analyses, the integrity of specific networks and tracts should be also evaluated (Bede, 2017; Tahedl, Tan, Chipika, et al., 2023). The shift from focality to circuitry has been repeatedly emphasized in ALS (Bak & Chandran, 2012; Bak et al., 2001; Grossman et al., 2008) and is consistent with the observation that specific neuropsychological functions are mediated by multisynaptic networks with distinct cortical and white matter components, many relayed through specific subcortical nuclei and sometimes modulated by cerebellar afferents (Bede et al., 2018; Bonelli & Cummings, 2007). The concomitant degeneration of interconnected gray matter regions and their association fibers is demonstrated by the ALS–FTD groups where the cortical degeneration of Broca's and Wernicke's areas is accompanied by the interconnecting arcuate fibers. While only supratentorial language regions were evaluated in this study, it is increasingly recognized that the cerebellum also plays a physiological role in language. Cerebellar pathology is well characterized in both ALS and primary lateral sclerosis (PLS) and has been confirmed by a series of radiology and postmortem studies (Bede, Chipika, et al., 2021; Chipika et al., 2022; Finegan et al., 2022) and also recognized in FTD (McKenna, Chipika, et al., 2021). From a methodological perspective, the value of running complementary structural functional analyses is well illustrated by this study. fMRI has been extensively applied toI ALS cohorts generating valuable academic insights (Proudfoot et al., 2018). Early fMRI studies in ALS were paradigm‐based studies focusing on motor regions, which were increasingly superseded by resting‐state studies, and more recently a series of elegant motor imagery studies have also been published. The benefit of rs‐fMRI lies in its ability to appraise connectivity along complex multisynaptic networks, but the potential confounds of underlying vascular pathology, vasoactive, and psychoactive medications have to be taken into account. This notion is supported by our findings of left hemispheric FC reduction between the endpoints of the frontal aslant track in cognitively unimpaired ALS patients, where traditional tractography did not detect white matter alterations. This demonstrates the benefit of evaluating a panel of structural and FC indices in motor neuron diseases as they may have divergent sensitivity to detect network degeneration (Abidi et al., 2021, 2020; Meier et al., 2020; Tahedl, Tan, Chipika, et al., 2023; Tahedl, Tan, Shing, et al., 2023; Trojsi, Di Nardo, Caiazzo, et al., 2020). SC is typically appraised based on diffusion data sets, which have their own caveats particularly around regions of crossing fibers. The most commonly evaluated diffusivity measure is FA, but as demonstrated in our study, it may have a limited detection sensitivity compared to other measures such as RD. In our data set, with the exception of the SLF, RD identified more significant group differences than FA. Novel diffusion models and high‐angular‐resolution DWI are increasingly implemented in ALS and FTD imaging as they are more likely to overcome the challenges associated with crossing fibers (Barritt et al., 2018; Broad et al., 2019; Trojsi et al., 2013).

Even though extrapyramidal, cerebellar, and frontotemporal deficits and their corresponding radiological changes are now well characterized in ALS (Abidi et al., 2022; Chipika, Finegan, et al., 2020; Christidi et al., 2023; Feron et al., 2018; Lulé et al., 2020; Trojsi et al., 2013; Trojsi, Di Nardo, Siciliano, et al., 2020), clinical trials continue to exclusively focus on motor function, respiratory measures, and survival as primary outcome measures. As demonstrated by this and other studies, a considerable radiological, pathological, and clinical overlap exist between ALS and FTD, and motor function is just one of the facets of ALS. There seems to be a compelling argument to also evaluate, monitor, and appraise extra‐motor involvement in clinical trials including executive dysfunction, memory, and language (Christidi et al., 2018). There is also a possibility that existing clinical trials in ALS may suffer from an inherent selection bias toward patients with no or limited cognitive impairment given the importance of comprehending, processing, and weighing important information.

The involvement of Broca's area, FAT, IFO, and UF in bvFTD is consistent with the pathological heterogeneity of this clinical phenotype. It is also noteworthy that many of our study groups exhibit marked bilateral changes and the pathology of relatively few ROIs was limited to the left hemisphere. From an ALS perspective, our data also highlight that *C9orf72* mutations are not the sole determinants of extra‐motor change. FAT RD is increased bilaterally in both ALS–FTD cohorts irrespective of *C9orf72* status and more interestingly, only the *C9orf72*‐negative ALS–FTD group demonstrates bilateral uncinate and right ILF involvement. This is well in line with other ALS and ALS–FTD imaging studies where cohorts who tested negative for *C9orf72* hexanucleotide repeat also demonstrate marked cortical, suborbital, and white matter pathology (McKenna, Tahedl, et al., 2021; Omer et al., 2017; Westeneng et al., 2016). With the emergence of antisense oligonucleotide therapies, genotype‐associated changes are of particular interest in both ALS and FTD (Li Hi Shing, McKenna, et al., 2021; McKenna et al., 2022; Nigri et al., 2023). Cluster analyses of unselected patient cohorts often revealed subpopulations of patients with particularly severe frontotemporal or cerebellar change (Bede et al., 2022; Dukic et al., 2022; Tan et al., 2022). While *C9orf72* hexanucleotide repeats in ALS are classically associated with marked frontotemporal dysfunction, a series of studies have highlighted that extra‐motor involvement is not unique to the *C9orf72* genotype (Westeneng et al., 2016). Our study focused on the most common ALS–FTD phenotypes, but there is an increasing recognition of language deficits in other, non‐ALS MNDs, such as PLS, spinal and bulbar muscular atrophy, or post‐polio syndrome (de Vries et al., 2019; Finegan et al., 2019, 2021; Li Hi Shing et al., 2019; Li Hi Shing, Lope, et al., 2021; Pradat et al., 2020).

While we have used standard descriptive statistics in this study, the identification of brain regions and imaging indices that reliably distinguish disease phenotypes is of increasing practical relevance. A variety of machine learning and *z*‐score‐based models have been recently applied to imaging data sets both in FTD and ALS to categorize individual subjects into clinically relevant diagnostic subgroups (Bede, Murad, & Hardiman, 2021; Behler et al., 2022; Grollemund et al., 2019; McKenna et al., 2022; McKenna, Tahedl, et al., 2021; Tahedl et al., 2021). Even though the classification accuracy of these models varies significantly at present, it is conceivable that multimodal MRI data will be used in the future for precision diagnostic and prognostic classification (Bede, Murad, Lope, et al., 2021; Schuster et al., 2016, 2017).

This study is not without limitations. It merely assesses established language‐associated tracts in a cross‐sectional design. We have no postmortem data to contrast our in vivo imaging findings with postmortem proteinopathy patterns. Similarly, no accompanying CSF or serum biomarkers were collected at the time of scanning. Notwithstanding these limitations, our results demonstrate the involvement of language‐associated tracts and networks in patient populations along the ALS–FTD spectrum.

## 5 CONCLUSION

Language‐associated tracts and networks are not only affected in language‐variant FTD phenotypes, but also in ALS and bvFTD. Accordingly, language deficits should be routinely screened for early in the course of ALS and bvFTD irrespective of *C9orf72* status. The social, management, and quality‐of‐life implications of language deficits and their potential impact on clinical trial participation should be specifically studied in large prospective studies.

## AUTHOR CONTRIBUTIONS

**Marlene Tahedl**: Formal analysis; methodology; writing—original draft. **Ee Ling Tan**: Formal analysis; investigation; methodology; writing—original draft. **Rangariroyashe H. Chipika**: Conceptualization; data curation. **Jasmin Lope**: Formal analysis; investigation; methodology; writing—original draft. **Jennifer C. Hengeveld**: Formal analysis; investigation. **Mark A. Doherty**: Formal analysis; investigation. **Russell L. McLaughlin**: Formal analysis; investigation. **Orla Hardiman**: Conceptualization; investigation. **Siobhan Hutchinson**: Conceptualization; data curation; investigation. **Mary Clare McKenna**: Conceptualization; data curation; investigation. **Peter Bede**: Conceptualization; formal analysis; investigation; writing—original draft.

## CONFLICT OF INTEREST STATEMENT

The authors declare no conflicts of interest.

### PEER REVIEW

The peer review history for this article is available at https://publons.com/publon/10.1002/brb3.3250.

## Data Availability

Additional information on data processing pipelines can be requested from the corresponding author. Individual‐patient clinical and neuroimaging data cannot be made available due to institutional regulations and departmental policies.
